# Is age and not antiretroviral therapy the strongest risk factor for chronic pain in HIV-infected population?

**DOI:** 10.1186/s12879-021-05776-7

**Published:** 2021-02-01

**Authors:** Marcin Kowalski, Andrzej Horban, Bartosz Slomka, Karen Shahnazaryan, Witold Rongies

**Affiliations:** 1 Polish Medical Air Rescue, Clinical Governance Department, Warsaw, Poland; 2 Department of Adults’ Infectious Diseases, Medical Faculty, Medical University of Warsaw, Warsaw, Poland; 3 Department of Rehabilitation, Public Central Teaching Clinical Hospital University Clinical Center, Medical University of Warsaw, Warsaw, Poland; 4 Department of Rehabilitation, Medical Faculty, Medical University of Warsaw, Warsaw, Poland

**Keywords:** HIV, AIDS, Pain, Opioids, Antiretroviral treatment

## Abstract

**Background:**

Chronic pain in HIV-infected patients on effective antiretroviral therapy (ART) limits patients’ normal functioning both somatically and psychologically. The current state of knowledge on the topic is insufficient, with the underlying causes of this pain unexplained. Therefore we analyzed the frequency and factors associated with chronic pain in HIV-infected patients on ART.

**Methods:**

We conducted a prospective, survey study, including consecutive HIV-infected patients under specialist care at the HIV Outpatient Clinic of the Hospital for Infectious Disease in Warsaw between February 2014 and December 2016. During their routine visit all patients who agreed to participate in the study were surveyed using a study questionnaire. For all patients reporting any pain the Brief Pain Inventory (BPI) form and Douleur Neuropathique 4 Questions form (DN4) were completed. Data on history and current ART and laboratory measurements were obtained from electronical database. Chi-squared and Kruskal-Wallis tests were used for group comparison. The potential factors associated with chronic pain were identified via logistic regression models.

**Results:**

In total 196 HIV-infected patients were included in the study, 57 (29,1%) of them reported chronic pain. The reported pain was mostly (75%) limited to a single area of the body. In univariable logistic regression model the odds of chronic pain were significantly higher with increasing age (OR 1.36 [95%CI:1.17–1.58]), time under specialist care (OR 2.25 [95%CI:1.42–35.7]), time on ART (OR2.96 [95%CI:1.60–5.49]), previous ART with zidovudine (OR 2.00[95%CI:1.06–1.55]) and previous treatment with ddI, ddC or d4T (OR4.13 [95%CI:1.92–8.91]). Homosexual route of HIV infection as compared to injecting drug use was decreasing the odds of chronic pain (OR0.33 [95%CI: 014–0.75]). In multivariable analyses, adjusting for all above the only factor associated with chronic pain was age (OR1.28 [95%CI:1.06–1.55]).

**Conclusions:**

The prevalence of chronic pain in the studied population of HIV-infected Polish patients was high. The only risk factor for chronic pain identified was age. With ageing HIV population it is therefore imperative to develop cooperation protocols for specialist HIV treatment clinics, pain treatment clinics, and rehabilitation units.

## Background

As a result of introducing combination antiretroviral therapy (cART) for HIV-1-positive patients their survival has been improved to the level of that in the general population [1, 2]. One of the effects of this phenomenon is the increased prevalence and early onset of non-infectious co-morbitidies (particularly cardiovascular disease, chronic kidney disease, type 2 diabetes mellitus, and cancer) in HIV-infected individuals [3–6]. At the same time, we are observing accentuated aging in HIV-infected people, which is associated mainly with increased immune activation, impaired regulatory functions, as well as direct effects of HIV replication in tissues and organs. One important question that has not been answered yet is whether or not osteoarthritis is more common and/or occurs at a younger age in this population in comparison with the general population. If so, this may have a significant impact on the overall benefit of cART [7–11]. Accentuated aging and early onset of selected non-infectious diseases in HIV-1-positive adults result in an increasing prevalence of pain (of various nature) in this population. It is chronic pain that constitutes a particularly big challenge for the primary healthcare team, as it is believed to detrimentally affect the patients’ personality, their mental balance, and their ability to perform their social and professional roles [10, 12].

Chronic pain is common among HIV-infected patients and has an impact on quality of life and antiretroviral adherence. The current state of scientific research in this field is insufficient, and the causes of pain phenomena remain largely unexplained. However, there is scientific evidence that chronic pain is associated with an increased incidence and level of depression, which may be an additional reason hindering the treatment of HIV-infected people, especially in the aspect of therapeutic adherence. Emotions, combined with a pain that commands the patient’s attention, make the experience even harder to bear. Not uncommonly, the situation is made worser by problems at work due to an inability to perform the required tasks, which leads either to financial problems or experiencing the lack of professional fulfillment [12–14].

### Objectives

The purpose of this study was to determine the prevalence of chronic pain in the HIV-infected population remaining under continuous specialist care, as well as the underlying causative factors of such pain.

## Patients and methods

We performed prospective, survey study including consecutive HIV-infected patients under specialist care at the HIV Outpatient Clinic of the Hospital for Infectious Disease in Warsaw between February 2014 and December 2016, focused on the presence or absence of the chronic pain. Study design, performance and analyses were performed in accordance with STrengthening the Reporting of OBservational studies in Epidemiology (STROBE) guidelines (https://www.bmj.com/content/335/7624/806).

The inclusion criteria for this study were the age of 18 years or older and a documented HIV-1 infection, as well as a written informed consent. Any mental condition, diagnosed based on the available clinical data (history-taking or the available medical records), was an exclusion criterion. Chronic pain was defined as pain lasting a minimum of 6 months. The data on the subject’s sex, age, route of HIV infection, age at the time of registration at a specialist clinic for HIV/AIDS-positive patients, laboratory test results (CD4+ cell count, HIV viral load), duration of specialist care (in years), and history of antiretroviral therapy (ART) (yes or no) were obtained from the clinic’s electronic database (Table 1). All patients reporting any pain were additionally asked to fill in the Brief Pain Inventory (BPI) form and were subject to a brief examination performed by a physician who afterwards completed a DN4 Douleur Neuropathique en 4 Questions (DN4) form.

**Table 1:** Study group characteristics and selected clinical parameters that may affect the occurrence of pain in HIV-infected individuals

Study group parameters (*n* = 196)	Median	Q1	Q3
Age of subjects at the time of their registration at SC (years)	31.9	26.8	39.9
Age of subjects at study inclusion (years)	41.0	34.4	49.0
BMI prior to study inclusion (kg/m^2^)	23.6	21.3	25.9
BP prior to study inclusion: systolic/diastolic (mm Hg)	136/88	127/80	145/91
ART duration (years)	4.3	2.5	10.9
CD4+ cell count at registration at SC (cells/mcL)	350	192	526
CD4+ cell count prior to study inclusion (cells/mcL)	550	424	704
CRP levels at registration at SC (IU)	5.0	5.0	9.0
CRP levels prior to study inclusion (IU)	6.0	5.0	9.0
Serum HGB at registration at SC (g/dL)	14.2	12.8	15.1
Serum HGB prior to study inclusion (g/dL)	15.0	13.9	15.9

*ART* Antiretroviral therapy, *BMI* Body mass index, *BP* Blood pressure, *CRP* C-reactive protein, *HGB* Hemoglobin, *IU* International units, *Q* Quartile, *SC* Specialist HIV/AIDS clinic

The general information form contained questions on the history of any pain over the previous week, mean duration, date of onset, frequency, and any help by physicians other than an infectious disease specialist (particularly those specializing in pain therapy) in dealing with the pain. Subsequent questions addressed the use of any psychoactive drugs or other drugs that could affect the perception of pain, as well as cART adherence (including the frequency of missing a dose).

The subjects who reported pain in the initial questionnaires went on to complete the BPI – Short Form [15]. Some of the questions in this 9-item questionnaire are about the intensity of pain felt at the moment and within the previous 24 h, quantified in an 11-point numerical rating scale, ranging from 0 to 10, where 0 indicates no pain and 10 – the worst pain imaginable. In addition, all respondents were asked to mark the approximate location of their pain on a diagram of the human body. The questionnaire also asks about treatments or medications used for the pain and asks the respondent to rate the effectiveness of these treatments or medications on a scale from 0 to 100% (in 10% increments), where 0% indicates no relief and 100% – complete pain relief. The questionnaire also assesses the extent to which the pain interferes with selected aspects of everyday life (normal work, general activity, mood, walking ability, relations with other people, sleep, and enjoyment of life) with the answer choices ranging from zero (no interference) to 10 (complete interference).

### Statistical analyses

In order to compare the study groups the Chi-squared and Kruskal-Wallis tests were used in statistical analysis. The level of significance was established at *p* < 0.05 All variables were tested for distribution and all presented skewed distribution, therefore non-parametric tests were used in analyses. The potential factors associated with chronic pain were identified by logistic regression analyses. Variables tested in univariable models were: sex; route of HIV infection; duration of specialist care (years); age at study inclusion; body mass index (BMI), systolic and diastolic blood pressure, hemoglobin levels, C-reactive protein levels, immunological parameters (absolute CD4+ and CD8+ cell counts, CD4+ cell percentage), HIV RNA levels, anti-HCV antibodies, total anti-HBc antibodies, VDRL test result (positive, negative, inconclusive); previous ART; previous treatment with zidovudine (AZT) and/or a ‘D’ drug (ddI, ddC, d4T); ART duration (years); achieved undetectable viral load (< 50 HIV RNA copies/mL following ART initiation) (yes or no); viral rebound (≥50 HIV RNA copies/mL) after complete suppression (yes or no). Moreover, the analyzed variables included the most recent (prior to study inclusion) levels of hemoglobin, C-reactive protein, and immunological parameters (CD4+ and CD8+ cell counts, CD4+ cell percentage). All variables tested as significant in univariable model (*p* < 0.01) were included in multivariable model. All statistical analyses were conducted with the use of SAS version 9.3 (SAS Institute, Cary, NC).

## Results

A total of 196 patients were included in the study, 57 (29.1%) reported chronic pain. Patients’ median age was 41.1 years. Men constituted 82.6% of the study population, with 41 subjects (20.9%) having contracted HIV through heterosexual contact, 90 (45.9%) through homosexual contact, and 37 (18.9%) through the use of intravenous drugs. The subjects who had contracted HIV in a different way constituted 4.1%, and those who contracted the infection via an unknown route constituted 10.2% of the study population. All patients included in the study had been under specialist care at the HIV Outpatient Clinic of the Hospital for Infectious Disease in Warsaw, Poland at the time of enrollment. The individual subjects were included in the study in the order they presented at their infectious disease specialist’s office. Table 1 shows detailed patients’ characteristics.

Experiencing pain in the week prior to study inclusion was reported by 96 subjects (48.9% of the study group). The mean duration of pain was characterized as “several seconds” by 3 subjects (3.1%), “several minutes” by 28 subjects (28.9%), “several hours” by 51 subjects (52.6%), whereas “continuous pain” was reported by 13 subjects (13.4%). Out of the subjects reporting pain, 57 (59.4%) identified the onset of symptoms as over 6 months before study enrollment.

### Location of chronic pain (*n* = 57)

The part of the body most commonly reported to be affected by chronic pain were the lower limbs (24 subjects; 42.1%), followed by the upper limbs (15 subjects; 26.3%), back and lumbosacral region (13 subjects; 22.8%), head (11 subjects; 19.3%), abdomen (8 subjects; 14.0%), chest (4 subjects; 7.0%), and other regions (3 subjects; 5.3%). Generalized pain was reported by 3 subjects (4.3%). Forty-three subjects (75.4%) reported pain limited to a single region of the body, 12 subjects (21%) reported pain limited to two regions, and 2 subjects (3.5%) reported pain limited to 4 regions.

### Pain intensity score in a numerical rating scale

The subjects who declared chronic pain (*n* = 57) rated pain intensity in a numerical rating scale (NRS). According to the established standard for this scale, the score of 1–4 points indicated mild pain, 5–6 points indicated moderate pain, and 7–10 points indicated severe pain. Assessed over the period of the previous 24 h, the intensity of pain was rated as mild, moderate, or severe by 19 (33.3%), 24 (42.1%), and 14 (24.6%) subjects, respectively, when assessing their pain at its worst; by 47 (82.5%), 7 (12.3%), and 3 (4.2%) subjects, respectively, when assessing their pain at its least; and by 44 (77.2%), 7 (12.3%), and 6 (10.5%) subjects, respectively, when assessing their pain on the average. At the time of completing the questionnaire, 48 subjects (85.7%) rated their pain as mild, 4 subjects (7.1%) – as moderate, and 4 subjects (7.1%) – as severe.

### Comparison of patient characteristics between the chronic pain group (*n* = 57) and no chronic pain group (*n* = 139)

Both study groups were statistically comparable with respect to sex (22.8% of women in the chronic pain group vs. 15.1% in the no chronic pain group; *p* = 0.216), the presence of anti-HCV antibodies (28.0% vs. 17.3%, respectively; *p* = 0.214), total HBcAb titers (24.6% vs. 24.5%; *p* = 0.962), positive Venereal Disease Research Laboratory (VDRL) test (14.0% vs. 15.8%; *p* = 0.509), the use of psychoactive drugs over the evaluated period (8.8% vs. 5.5%; *p* = 0.519).

The median lymphocyte counts at the time when the subjects started to receive specialist care and at the study inclusion were also comparable between the two groups. In the group of those reporting pain, the median CD4+ cell count at the time when they started receiving specialist care was 348 cells/mcL (vs. 350 cells/mcL in the no-pain group; *p* = 0.761), with 25% of the pain-reporting subjects having CD4+ counts lower than 189 cells/mcL (vs. < 196 cells/mcL in the no-pain group). The most recent (prior to study inclusion) median CD4+ cell counts were higher at 516 cells/mcL and 563 cells/mcL, respectively (*p* = 0.256).

At study inclusion, 184 subjects were receiving ART (55 in the pain group and 127 in the no pain group). The two groups were comparable in terms of ART rates (98.2% vs. 92.1%, respectively; *p* = 0.185) and initial viral suppression (98.2% vs. 99.2%; *p* = 0.517). Poor cART adherence (based on an initial questionnaire) was reported by 38.2% of subjects with chronic pain and by 30% of those without chronic pain (*p* = 0.306).

The two groups differed significantly in terms of age at study inclusion (with the median age of 45.3 years in the pain group vs. 39.6 years in the no pain group; *p* = 0.0002); median duration of specialist care (10.8 years vs. 4.9 years, respectively; *p* = 0.0008), median nadir CD4+ cell counts (168 cells/mcL vs. 253 cells/mcL), median duration of ART (8.5 years vs. 3.4 years; *p* = 0.0046), viral rebound after complete suppression (5.1% vs. 38.3%; *p* = 0.018), as well as previous treatment with zidovudine (44.6% vs. 30.5%; *p* = 0.063) and ‘D’ drugs (33.9% vs. 11%; *p* = 0.0004) (Table 2).

**Table 2:** Univariable and multivariable logistic regression analyses of variables among HIV-infected studied patients

	univariable analysis			multivariable analysis			
Variable	OR	95% CI	*P* value	OR	95% CI		*P* value
Age at study inclusion (per 5-year increment)	1.36	1.17–1.58	< 0.001	1.28	1.06	1.55	0.0089
Nadir CD4+ count (per 100-cell/mcL increment)	0.81	0.66–1.01	0.06	1.16	0.88	1.53	0.286
Duration of specialist care (per 10-year increment)	2.25	1.42–3.57	< 0.001	0.73	0.22	2.48	0.617
Duration of ART (per 10-year increment)	2.96	1.60–5.49	< 0.001	1.87	0.37	9.46	0.448
HIV risk group							
Heterosexual contact vs. IDU	0.54	0.21–1.38	0.362	0.63	0.18	2.19	0.668
Homosexual contact vs. IDU	0.33	0.14–0.75	0.005	0.46	0.15	1.45	0.168
Other vs. IDU	2.19	0.45–10.5	0.071	1.33	0.23	7.51	0.384
Unknown vs. IDU	0.56	0.18–1.79	0.528	0.59	0.14	2.45	0.633
Viral rebound following complete suppression	2.45	1.30–4.59	0.005	1.32	0.58	3.01	0.503
Previous treatment with AZT	2.00	1.06–3.80	0.0336	0.67	0.25	1.77	0.422
Previous treatment with ‘D’ drugs (ddI, ddC, d4T)	4.13	1.92–8.91	< 0.001	2.11	0.69	6.42	0.188

*AZT* Zidovudine, *IDU* Intravenous drug use

The two groups also differed significantly in terms of the route of HIV infection. The chronic pain group was characterized by higher rates of individuals who contracted HIV through intravenous drug use (28.1% vs. 15.1%), heterosexual contact (21.0% vs. 14.8%), and in other ways (8.8% vs. 2.2%); whereas the no chronic pain group was characterized by higher rates of individuals who contracted HIV through homosexual contact (36.7% vs. 31.6%). The route of infection was characterized as unknown by comparable proportions of subjects from both groups .

### Logistic regression analysis of factors associated with chronic pain

Univariable analyses showed that the epidemiological factors associated with the development of chronic pain were: the route of HIV infection, age at study inclusion, and specialist care duration (in years)**.** The odds of developing chronic pain increased by 36% with each 5-year increase in the age of subjects at study inclusion and by 8% with each 10-year increment in the duration of specialist care. Analysis of the routes of HIV infection yielded a statistically significant result when the intravenous drug use (IDU) route was adopted as reference, and the *p*-value for effect was 0.0307. Compared with the IDU subgroup, the subgroups infected through the heterosexual, homosexual, and unknown routes showed lower chances of developing chronic pain. However, statistical significance was demonstrated only while comparing the homosexual and IDU routes, with the subjects infected through homosexual contact showing a 67% lower risk of developing chronic pain in comparison with those infected through IDU. The subgroup that reported HIV infection through a different route showed significantly (over two-fold) higher risk of developing chronic pain in comparison with the IDU subgroup Table 3.

**Table 3:** Comparison of baseline characteristics of HIV- infected patients with and without chronic pain

Variables	Patients with chronic pain *N* = 57	Patients with no pain *N* = 139	
	Median	Median	*p* value
Duration of specialist care (in years)	10.8	4.9	0.0008
Age at enrollment (years)	45.3	39.6	0.0002
Duration of ART (years) (N = 196)	8.5	3.4	0.0046
Values of Parameter ​​when included in specialist care			
Hemoglobin (g/dl)	14.0	14.3	0.126
Creative protein C	5	5	0.342
CD4+ lymphocyte count	348	353	0.761
CD8+ lymphocyte count	916	872	0.693
CD4+ percentage	30.0	26.0	0.363
HIV RNA (copies/ml)	18,100	33,915	0.151
Last available values before inclusion in the study			
BMI	23.5	23.6	0.707
Systolic blood pressure	125.6	125.0	0.826
Diastolic blood pressure	78.1	78.0	0.766
Hemoglobin (g/dl)	14.8	15.1	0.578
C-Reative Protein (CRP)	7.0	6.0	0.147
Lowest nadir CD4+ count	168	253	0.0206
CD4+ lymphocyte count	516	563	0.256
CD4+ lymphocyte percentage	40.0	41.0	0.294
CD8+ lymphocyte count	731	735	0.892

Ultimately, 184 subjects were included in the multivariable regression model. Those subjects who were not receiving any ART at the time of inclusion into the study were excluded from this analysis due to the fact that various aspects of ART were to be analyzed in this model. After adjustment for all factors significant in univariable analyses the only factors significantly associated with chonic pain was age. The odds ratio per 5-year increase in age was OR = 1.28, with the 95%CI of 1.06–1.55 (*p* = 0.0089). This means that when comparing two individuals with the same baseline characteristics (i.e. sex, route of HIV infection, nadir CD4+ cell count, specialist care duration (years), ART duration (years), previous use of AZT and ‘D’ drugs, and ART adherence (based on the presence or absence of viral rebound following complete suppression), the 5 years older individual had a 28% higher chance of developing chronic pain. From an individual perspective, this result indicated that with each 5-year increase in the patient’s age the risk of developing chronic pain was higher by 28%.

## Discussion

This study, conducted in a population of HIV-infected patients on ART showed high rates of pain, but lower than reported elsewhere rates of chronic pain. One half of the patients in the study population reported pain at the moment of study inclusion, whereas one-third reported chronic pain. In order to obtain a representative sample of all HIV positive patients in our clinic the study included consecutively all patients admitted for regular out-patient’s visits without any preselection, except mental diseases and no informed consent.

A systematic review by Parker et al. regarding pain in HIV-infected patients showed the rates of chronic pain (defined as pain lasting more than 3 months) ranging from 54 to 83%. That review included 61 studies published between 1993 and 2011, and thus included also the studies conducted in treatment-naïve populations, populations undergoing potentially neurotoxic therapies, as well as populations receiving low-toxicity cART [16]. In comparison, the proportion of chronic (> 3 month long) pain reported by Robbins et al. in a 2011 study in a group of 254 HIV-infected patients in Thailand was 22% [17]. Another study (by Uebelacker et al.), based on the data collected in a group of 238 patients in the United States in the period 2012–2013, showed that chronic pain of over 6 months occurred in 53% of the study population [18]. Lawson’s study, conducted in a population of 1050 patients in the United Kingdom in 2014, showed that as much as 50% of the study population reported pain lasting more than 3 months [19]. A recent study by Jiao et al. in a group of outpatients demonstrated chronic pain in 40% of the study population [20], whereas in a 2010 study by Aouizerat et al. pain was reported by 55% subjects, 67% of whom characterized it as frequent or nearly continuous [21].

In comparison with the studies quoted above, our study demonstrated comparable rates of reported pain, with somewhat lower rates of chronic pain. However we have performed our study in more recent years, when less toxic and more effective ART was available. We would like to emphasize that comparing different studies in terms of the rates of pain is very difficult, due to the considerable variations in the study populations. For instance, a population of Thai patients practically cannot be compared with any European patient population, due to their substantial differences, both genetic and cultural. Even comparisons between European and American studies are difficult, as American studies are frequently conducted in very specific populations, e.g. in a population of social outcasts. Another obstacle in comparing the results of different pain-related studies is the lack of a universally accepted definition of chronic pain [22–26]. For example, in an American study by Miaskowski et al., as well as in our study, chronic pain was defined as pain lasting over 6 months. However, the proportion of the study population reporting pain in that American study (in contrast to that in our study) was very high at 90%. In light of the fact that the population analyzed in the American study had been recruited from the REACH cohort (constituting exclusively the homeless), any reliable comparison with our study is impossible.

The fact that the prevalence of chronic pain in our study was lower than that in the study by Lawson et al. [19] is most likely due to their defining chronic pain as pain lasting over 3 months.

Comparing the results of our study with the studies on chronic pain conducted in HIV-negative adults, chronic pain was considerably more common in the HIV-infected population. The 2006 study by Breivik et al. has the most similar study design to that of our study, as those authors had adopted the same definition of chronic pain as that in our study, and the study populations were comparable [27]. Breivik’s study involved several tens of thousands subjects from about a dozen European countries and Israel and showed that 19% of them suffered chronic pain (defined as pain lasting ≥6 months) [27]. A 2014 study by Kennedy et al. also observed chronic pain (defined as pain lasting > 3 months) in 19% of adults from a large sample of the general population in the United States [28]. However, a recent, 2016, literature review by Fayaz, regarding the general population of the United Kingdom showed higher rates of chronic pain ranging from 35 to 51% [29]. Like the study by Kennedy et al., the review by Fayaz defined chronic pain as pain lasting 3 months or more.

Another important aspect of our study involved determining the *severity of chronic pain.* In our study population the average pain intensity over the previous 24 h was rated as moderate or severe by one in five subjects (22.8%). This is a much lower proportion than that reported in other studies, already mentioned above. For instance, the study by Miaskowski conducted in a cohort of the homeless in San Francisco, showed 92% of the chronic pain cases to be of moderate to severe intensity [30]. Most of the 18 papers on the severity of chronic pain evaluated as part of the already mentioned systematic review by Parker et al. showed moderate to severe pain [16]. In comparison, in the study by Uebelacker et al. the vast majority (81%) of patients with chronic pain rated its average intensity over the previous week as moderate or severe [18]. In Lawson’s study, subjects rated the pain felt at that moment in an 11-point Visual Analogue Scale (VAS), and the median pain severity was 5 (i.e. moderate pain) [19]. The severity of chronic pain in HIV-negative populations can be compared, as before, with that in the 2006 study by Breivik et al., where most subjects rated the last pain they felt as moderate or severe (NRS was used, as in our study) [27].

### Limitations of the study

Comparing the individual studies in terms of pain severity also raises many doubts due to the differences in study methodologies. Comparison difficulties may be due to the differences in the questions on pain intensity, on the period when the pain was present (“right now”, “within the last 24 hours”, “within the last week”), and on the pain intensity “on the average”, “at its worst”, “at its least” in the given period. HIV-infected individuals in Poland may be subject to a greater stigmatization, particularly self-stigmatization. This, in turn, may lead to lowered expectations in terms of their quality of life and, in consequence, to underrating their pain intensity. The Stigma Index study clearly showed that in the populations of some Eastern European countries (Poland, Estonia, Moldova, Turkey, and Ukraine) stigmatization of HIV-infected individuals is greater than that in Western Europe [31]. In our opinion, future studies aiming to compare the prevalence of pain in HIV-infected populations in various countries should put more emphasis on evaluating the factors that affect the subjective assessment of pain as well as the unquantifiable causative factors responsible for the severity and nature of pain [31].

We have not identified older ART regimens to be independently associated with chronic pain. This may be related to a long time from use of this drugs. As indicated by randomised clinical trial by Arenas-Pinto et al. even in cases where neuropathy occurred it had a tendency to fade away with time [32].

## Conclusions

The prevalence of pain (including chronic pain) in the evaluated population of Polish HIV-infected patients was high. The most significant risk factor for the development of chronic pain in our study group was age, which constitutes a significant clinical and epidemiological problem, considering the aging of the HIV-infected population. It is crucial to develop standards for collaboration between specialist HIV treatment clinics and chronic pain treatment facilities as well as rehabilitation clinics and facilities.

## Data Availability

The datasets used and/or analysed during the current study are available from the corresponding author on reasonable request.

## Abbreviations

cART: Combination antiretroviral therapy
ART: Antiretroviral therapy
AUDIT-C: Alcohol Use Disorders Identification Test Consumption
BPI: Brief Pain Inventory
DN4: Douleur Neuropathique en 4 Questions
IDU: Intravenous drug use
NRS: A Numerical rating scale
